# Study Protocol for “Psilocybin as a Treatment for Anorexia Nervosa: A Pilot Study”

**DOI:** 10.3389/fpsyt.2021.735523

**Published:** 2021-10-20

**Authors:** Meg J. Spriggs, Hannah M. Douglass, Rebecca J. Park, Tim Read, Jennifer L. Danby, Frederico J. C. de Magalhães, Kirsty L. Alderton, Tim M. Williams, Allan Blemings, Adele Lafrance, Dasha E. Nicholls, David Erritzoe, David J. Nutt, Robin L. Carhart-Harris

**Affiliations:** ^1^Centre for Psychedelic Research, Department of Brain Sciences, Imperial College London, London, United Kingdom; ^2^OxBREaD Research Group, Department of Psychiatry, University of Oxford, Oxford, United Kingdom; ^3^School of Rural and Northern Health, Laurentian University, Sudbury, ON, Canada; ^4^Division of Psychiatry, Department of Brain Sciences, Imperial College London, London, United Kingdom

**Keywords:** psilocybin, anorexia nervosa, clinical trial, eating disorder, magnetic resonance imaging (MRI), electroencephalograph (EEG), public patient involvement (PPI), psychedelic-assisted therapy

## Abstract

**Background:** Anorexia nervosa (AN) is a serious and life-threatening psychiatric condition. With a paucity of approved treatments, there is a desperate need for novel treatment avenues to be explored. Here, we present (1) an overview of the ways through which Public Patient Involvement (PPI) has informed a trial of psilocybin-assisted therapy for AN and (2) a protocol for a pilot study of psilocybin-assisted therapy in AN currently underway at Imperial College London. The study aims to assess the feasibility, brain mechanisms and preliminary outcomes of treating anorexia nervosa with psilocybin.

**Methods:** (1) PPI: Across two online focus groups, eleven individuals with lived experience of AN were presented with an overview of the protocol. Their feedback not only identified solutions to possible barriers for future participants, but also helped the research team to better understand the concept of “*recovery”* from the perspective of those with lived experience. (2) Protocol: Twenty female participants [21–65 years old, body mass index (BMI) 15 kg/m^2^ or above] will receive three oral doses of psilocybin (up to 25 mg) over a 6-week period delivered in a therapeutic environment and enveloped by psychological preparation and integration. We will work with participant support networks (care teams and an identified support person) throughout and there will be an extended remote follow-up period of 12 months. Our two-fold primary outcomes are (1) psychopathology (Eating Disorder Examination) across the 6-month follow-up and (2) readiness and motivation to engage in recovery (Readiness and Motivation Questionnaire) across the 6-week trial period. Neurophysiological outcome measures will be: (1) functional magnetic resonance imaging (fMRI) brain changes from baseline to 6-week endpoint and (2) post-acute changes in electroencephalography (EEG) activity, including an electrophysiological marker of neuronal plasticity.

**Discussion:** The results of this pilot study will not only shed light on the acceptability, brain mechanisms, and impression of the potential efficacy of psilocybin as an adjunct treatment for AN but will be essential in shaping a subsequent Randomised Control Trial (RCT) that would test this treatment against a suitable control condition.

**Clinical Trial Registration:** identifier: NCT04505189.

## Introduction

“*In the core you are always yourself, but during an eating disorder you lose yourself… looking back I was just a hollow kind of body…you lose yourself in the process but you find yourself again and you also create new…I reinvented myself but then I also reconnected with what I always was.”* (Focus group 2, A1).

Anorexia nervosa (AN) is a serious psychiatric condition. The Diagnostic and Statistical Manual of Mental Disorders (DSM-5) (1) defines AN by severe nutritional restriction, an intense fear of gaining weight, and a disturbance of one's bodily self-perception. Prevalence rates are highest in females (approximate lifetime prevalence of 1.2–2.2%), and onset is most common in adolescence or early adulthood (2, 3). AN is not a Western-bound illness, nor is it constrained to those of a particular age, gender, culture or ethnicity (4–7).

AN is the most fatal of all psychiatric conditions. Mortality rates are 11.6 times higher than the general population (8). Of these premature deaths, 25% result from suicide, with the reported suicide rate in AN up to 56 times that of the global average (9). Treatment dropout rates in adult patients are also staggeringly high with 20 to 50% of inpatients, and up to 70% of outpatients ending treatment prematurely (3, 10–12). Fewer than half of those diagnosed reach remission after specialist treatment (13, 14) and for 20–30%, AN is a life-long condition (15, 16)^1^. Despite these high mortality and low remission rates, AN research remains severely underfunded with <1% of (the already limited) mental health research funding in the UK allocated to eating disorders, compared with up to 9% for depression (13, 18, 19).

In recent years there has been a surge of interest in the therapeutic use of classic psychedelic drugs; a class of drugs including lysergic acid diethylamide (LSD), psilocybin (the principal psychedelic molecule in magic mushrooms), and dimethyltryptamine (DMT, the principal psychedelic molecule in ayahuasca). There is a growing list of clinical trials highlighting the potential efficacy of psychedelic therapy across conditions (20) including various addictions (21–23), obsessive-compulsive disorder (24), depression (25–29), and end-of-life anxiety (30–33).

The primary receptor-level mechanism of action of classic psychedelics is thought to be via agonism of the serotonin 2A (5-HT_2A_) receptor. This is understood to induce a spike to wave decoherence that leads to an associated acute “entropic brain state” whereby the spontaneous activity of neural ensembles becomes dysregulated. This 5-HT_2A_ stimulation also induces a hyper-plastic state and a speculated relaxation of the precision weighting of implicit predictive mechanisms. Within the right context, this is thought to promote rapid and deep learning and open a window of opportunity for meaningful psychological transformation (34–37). There is also now an increasing understanding that it is the *quality* rather than the *intensity* of the acute experience that is predictive of long-term therapeutic outcomes (21, 31, 33, 38–40) with growing recognition for the importance of acute emotional-breakthroughs (41, 42).

In AN, aberrant serotonergic activity (43) and reduced synaptic plasticity-related brain-derived neurotrophic factor (BDNF) serum concentrations (44) are thought to contribute to the psychopathology. AN is also characterised by cognitive rigidity (45, 46) and experiential (47) or emotional avoidance (48, 49). Such traits have been demonstrated to change following a meaningful psychedelic experience where the experience of intense emotional states is an important part of the therapeutic process (25, 41, 50, 51). Based on both this neurobiological and psychological evidence, psilocybin assisted-therapy deserves consideration as a potentially novel therapeutic avenue for AN.

There is also preliminary evidence for the efficacy of psychedelics in the treatment of AN. A single case study from 1959 presents a female patient (Miss Henriette B…) for whom two doses of intravenous psilocybin induced “indisputable” therapeutic action (52). Most notable were the patient's acute insights into the perceived root causes of her symptoms, post-acute improvements in mood and well-being, and subsequent weight restoration. Following participation in ceremonial ayahuasca experiences, Lafrance and colleagues reported a reduction or cessation of ED symptoms, improvement in body perception, and increases in self-acceptance in qualitative reports from those with a diagnosis of an ED (53, 54). Finally, in a prospective study, Spriggs et al. (42) demonstrated reductions in depressive symptoms and increases in well-being 2 weeks after a self-initiated psychedelic experience in those reporting a lifetime diagnosis of an ED, with supportive evidence for a role for acute emotional breakthroughs in mediating this change.

These strands of evidence support an early phase clinical trial into the potential of psilocybin-assisted therapy to treat AN. We are not alone is this surmise (55). At the point of writing, there are three trials of therapy with psilocybin (NCT04052568, NCT04505189, NCT04661514) and one study of therapy with 3,4-Methylenedioxy methamphetamine (MDMA; NCT04454684) either currently underway or planned in this population. In summary this represents a novel application for the burgeoning field of psychedelic-assisted therapy, as well as a potential opportunity to address the dire need for new treatment options for AN. Here, we present the protocol for a trial of psilocybin as a treatment for AN that is taking place at the Centre for Psychedelic Research, Imperial College London. First however, we demonstrate how Public Patient Involvement (PPI) was incorporated into the development of the trial, and how this has been crucial in shaping how it will be conducted.

## Public Patient Involvement

PPI is research carried out *with* or *by* members of the public rather than *to* or *about* them (56, 57). There is growing recognition for the important role for PPI in producing relevant, transparent and accountable research (57, 58). While there is no indication that PPI has been widely utilised in psychedelic research to date, two other publications in this special issue of Frontiers Psychiatry emphasise the importance of PPI to this field [Bornemann (under review)^2^, (59)].

In preparation for this study, we conducted two online PPI focus groups (60). The aims of the focus groups were to (1) identify common concerns among individuals with AN regarding our trial design, (2) explore common barriers that individuals with AN face when engaging in treatment/research, (3) identify practical ways of addressing relevant barriers. Here we report the methods, outcomes and reflections from our PPI focus groups in accordance with the GRIPP2 reporting checklist (61).

### PPI Methods

Attendees were invited to take part through poster advertisements on social media. Two 2-hour focus groups were held online with different groups of attendees. The focus groups were facilitated by three members of the trial's research team: a lead facilitator (MS), a scribe (HD), and a psychiatrist for mental health support (KA or FM). Eleven females aged 19 to 35 (*M* = 25.36, *SD* = 4.80) in various stages of treatment/recovery from AN attended one of the two focus groups. Attendees were reimbursed for their time.

Each focus group started with a brief presentation outlining the study design. Attendees were then invited to ask questions and provide feedback. An open discussion was then facilitated which was loosely centred on one or both of two broad questions: (1) what blocks have you faced in treatment for anorexia? (Focus group 1) and (2) what does “recovery” mean to you? (Focus group 1 & 2). Following the focus group, attendees were also invited to speak to the lead facilitator one-to-one. In the focus groups, attendees were encouraged to develop ideas collaboratively, while the one-to-one calls gave each attendee a further opportunity to share more personal or sensitive reflections.

Following each focus group, key themes from the discussions were identified by the lead facilitator and a summary document was created that outlined these themes and the implications for the trial. All attendees were invited to provide feedback on the summary document from their group, as well as feedback on the session itself. Specific consent was obtained to use quotes included in these summary documents for future publications and public engagement.

Brent National Research Ethics Service (NRES) and the Imperial College Research Governance and Integrity Team (RGIT) were consulted about all proposed activities. As this investigation was classified as PPI, no specific ethical approval was required for this component of the project.

### PPI Results and Discussion

#### Barriers to Engagement

The most pertinent barrier to engaging in treatment identified by attendees was a focus on weight restoration. While authoritarian refeeding regimes can be a lifesaving necessity in AN treatment, it is felt that they overlook the importance of the psychological factors underlying these issues (62–64) which leaves patients feeling powerless, alienated from their bodies, and misunderstood (12, 65, 66). One focus group attendee reflected that previous treatments had left her feeling “*backed into a corner, I felt like an animal backed into a cage”* (Focus group 1, A5) while another described a disconnect that “*When the therapist is overly focused on a goal recovery weight, that doesn't correlate with feeling better!”* (Focus group 1, A1). Focus group attendees were reassured to know that, while we acknowledge that there is no definition of recovery from AN that does not include weight restoration (and negotiating this tension is a key part of the therapeutic process), our outcome measures do not focus on weight, but rather on the eating disorder psychopathology central to their distress. It was agreed that anxiety around weight checks will be minimised by ensuring that participants are aware of weight checks ahead of time, and that this information is being recorded primarily for safety and monitoring purposes as opposed to as an expected outcome.

Focus group attendees also identified a “reluctance to give up control” as being one of the key barriers that they would face in participating in a trial such as this. The idea of taking a drug that would induce a psychedelic experience felt particularly intimidating:

“*I've done so many bad things and whatever, but touching drugs, definitely would be scary…”* (waving hands for no; Focus group 1, A1).

In AN treatments, moving through significant “negative” emotions mid-treatment has been associated with improved outcomes (67). Psychedelic experiences can also be experienced as more challenging in the short term as one struggles to grapple with new insights (68). Focus group attendees were aware of the potential for this:

“*What do I do with the deluge that comes when the dam is broken?”* (Focus group 1, A2).

Importantly, attendees also suggested a number of steps to overcome this “giving up control” barrier. Firstly, providing adequate information on the safety and acute effects of psilocybin may help participants to feel more at ease. Secondly, building a trusting relationship between the participant and the guides was identified as pivotal. As stated by one attendee:

“*They know if they want to open their eyes that you will be sitting next to them with a hand to hold. Sometimes that is more important than information on how it works.”* (Focus group 2, A5).

Third, advanced practical planning around dosing days (e.g., meal planning, weight checking and accommodation) will increase trust and enable greater engagement with the therapy. We have added this as one of the of the focuses of participants' first preparation calls with their guide team (see section Psilocybin-Assisted Therapy for further information). Finally, attendees suggested that establishing good communication with support networks would help participants to feel that they have a plan for when the trial is over, which may create space for greater engagement. This is also consistent with our conjecture that support network involvement will be important for bridging the insights gained during the trial into real world behaviour changes, which has been incorporated into our trial procedures (see section Support Person Involvement for further information).

#### What Is “Recovery” From AN?

When posed with the question “what does “recovery” mean to you?,” both groups not only emphasised the importance of looking beyond diagnostic criteria, but also demonstrated the extent to which the definition of recovery varies from person to person and throughout the different phases of the recovery process. One consistent element however, was that both focus groups identified *rediscovery of identity* as a key component of recovery. AN is commonly regarded as an “egosyntonic” condition, where the disorder is central to one's identity and value system (64, 69–72). The existential threat of recovery as a process of generating a new self-identity (64, 71–73) was reflected in both focus groups:

“*So, I sort of get quite scared of the idea of trying to like, find those boundaries between me and the illness and wondering who I would be without it when I have lived my entire life building my sense of self while also having this there…what is life without it? That is my big black hole that puts me off going further in any kind of recovery or, like, actively engaging in recovery.”* (Focus group 1, A2).

“*You get so interwoven with the illness…it can be hard to separate the two out so in some ways it is easier to just rebuild yourself… until someone says to you, like, “who do you want to be? What identity do you want to have now? you can do whatever you want essentially,” there is no hope.”* (Focus group 2, A4).

Contrary to popular belief, physical appearance did not emerge as being at the core of what those with AN value about their condition. Instead, focus group attendees appeared cognisant of other functional roles their AN served and how difficult it would be to let them go:

“*You have no control over the things that are going on externally that are making you go crazy or hurting you or causing you pain, but oh yea damn you can really, you can control how much you love yourself and your nutritional intake in a heartbeat.”* (Focus group 2, A5).

“*…You've been holding on to it for so long, it was kind of that thing that keeps your head above the water so you wouldn't drown, and so letting go of that it really feels as if I am going to drown if I let go of that because I don't know who I am anymore.”* (Focus group 1, A4).

This is consistent with previous research in which those with AN report that AN offers a sense of security/control over emotions, a communication tool, and a form of mastery over their body and life (66, 69, 74–76). From this perspective, AN represents a predominant identification with the body-object (i.e., the *lived body for others*) over the body-subject (i.e., the *body experienced from within*) (76–78), where the body represents a “concretised metaphor” for an inner experience (76).

With this in mind, it is evident that a disconnect in the definition of recovery between patients with AN and clinicians could contribute to one of the most widely recognised features of AN: high ambivalence toward recovery and an intense fear of engaging in the recovery process^3^. In other words, those with AN feel trapped between logic and emotion; while they know they must eat to survive, they cannot help but feel fearful of it. This creates a state of ambivalence and a low motivation to engage in treatment that evokes anxiogenic states (73). Focus group attendees reflected on times when they had felt *willing* to recover, but *unable, unmotivated*, or *not ready*.

“*I wanted to recover, but I also didn't have the motivation, and I wasn't ready”* (Focus group 2, A1).

“*There was a willingness to get better, but I just couldn't”* (Focus group 2, A2)

Across previous studies, both clinicians and patients agree that increasing willingness and motivation to recover is an essential first step of any effective treatment (64, 70, 81). We therefore see this as an important outcome for this trial.

“*I think that for a lot of people, they chase this concept of recovery when it doesn't, for some people, you know there is no definition of what it means so it's kind of impossible to chase…integrating it and making peace with the fact that you have this and taking anxiety away from having those thoughts and just accepting it is something that I can see as being a good outcome of this kind of therapy.”* (Focus group 2, A4).

#### PPI Reflections

Each group was comprised of attendees with differing experience and knowledge of psychedelics and for some attendees, this was the first time they had heard of psychedelic-assisted psychotherapy. Because of this, the conversations did not centre as much on the specifics of the psychedelic experience *per se*, rather on aspects relevant to carrying out such a trial with people with AN. Importantly, we were able to identify key barriers to participation and develop strategies to help manage these. Additionally, the focus groups helped us to recognise the importance of trial participants being supported to develop their own understanding of recovery as a way of promoting motivation to engage in treatment. A study measure has subsequently been developed to capture this (the self-constructed “Recovery Interview”).

The use of focus groups not only fostered collaboration but had an unexpected positive impact on attendees who reported feeling a sense of community and understanding in recovery. Giving those with lived experience an opportunity to be heard was a rewarding experience for researchers and attendees alike. One limitation of these focus groups was the lack of diversity in the groups themselves. In future, we will endeavour to advertise PPI more broadly and will seek to actively engage with seldom-heard groups. A further limitation is that focus group attendees were largely ineligible for the study itself due to, for example, their age, location, stage of recovery, or current treatment. While a number of the attendees saw PPI as an alternative way of contributing to the study, some of the reflections shared may not be generalisable to study participants.

## Study Protocol: “Psilocybin as a Treatment for Anorexia Nervosa: A Pilot Study”

### Overview

Here we present the protocol for a pilot study that has been designed to assess the feasibility and preliminary outcomes of treating AN with psilocybin-assisted therapy. Additionally, the study aims to use Magnetic Resonance Imaging (MRI) and Electroencephalography (EEG) to examine the neuronal underpinnings of treatment with psilocybin in this group. Although pilot studies are often perceived as less impactful than larger scale RCTs, their potential influence in shaping the direction of future research should not be understated. It is the overarching goal of any pilot study to provide the optimal information needed to prepare for future studies, and they should therefore be held to the same level of methodological rigour as their larger-scale counterparts (82–84). A well-conducted pilot study will not only improve the resource efficiency of future large-scale studies but is also more ethical as it allows for a more data-driven approach to the development of key methods and outcomes (82). The outcomes of this study will sharpen the research focus and evaluate the utility and acceptability of study methods for implementation in a larger-scale (and therefore more costly) study.

### Methods and Analysis

#### Recruitment and Selection

We will include twenty completing participants with a DSM-5 diagnosis of AN in this study, with “completion” defined as completing the entire final study visit. Key entry criteria can be found in Table 1. We have endeavoured to match our pilot study inclusion criteria to those that might be used for any future larger studies to best represent the target population^4^.

**Table 1:** Key inclusion and exclusion criteria for the trial.

**Inclusion criteria**
Primary diagnosis of AN for more than 3 years
Current or past treatments have not been successful to maintain remission
21–65 years of age
Female at birth^a^
In the care of a UK GP and specialist ED team, and agree to have us maintain contact with both for the duration of the trial
Sufficient competency with the English language
BMI ≥ 15 kg/m^2^ and medically stable
Capacity to consent
Agreement to have us maintain contact with an identified support person
**Exclusion criteria**
Current or previously diagnosed psychotic disorder, significant history of mania, or evidence of a personality disorder
Immediate family member with a diagnosed psychotic disorder
Unstable physical condition. This includes rapid weight loss >2 kg in the prior month, abnormal serum electrolytes, raised cardiac enzymes, hepatic or renal dysfunction, abnormal QT interval prolongation (QTc above 470 ms)
Medical condition unsuitable for psilocybin, EEG or MRI
History or laxative abuse in the previous 3 months, or drug dependence in the previous 6 months
History of serious suicide attempts or presence of a suicide/serious self-harm risk at screening
Currently an inpatient
Blood or needle phobia
Positive pregnancy test or breastfeeding
Sexually active and lacking appropriate contraceptive means
No email access
Enrolled in another clinical trial of an investigational medicinal product (CTIMP) in the last 3 months

a *While the exclusion of males may appear at odds with the inclusive approach we wish to embody, we also recognise that the presentation and treatment needs of males with AN differ from those of females (85, 86). AN is also significantly less common in males [estimates of male EDs range from approximately 10 to 25% of cases (7, 87)], rendering recruitment of a balanced sample difficult*.

We will recruit via referrals from National Health Service (NHS) Trust specialist eating disorder teams, and through self-referrals from advertisements and word of mouth. Regardless of recruitment method, all participants will be required to be under the care of a specialist eating disorders service to participate, and we will contact their General Practitioners (GP) and eating disorder service to confirm eligibility.

GPs and eating disorder services will also be asked to confirm any medications their patient is currently taking. Patients with AN are often prescribed serotonergic medications such as selective serotonin reuptake inhibitor (SSRI) or atypical antipsychotics (e.g., quetiapine) to manage co-morbid depression or anxiety, but with limited efficacy in treating AN symptomology (88). As serotonergic medications attenuate the acute psychological effects of psychedelics (89) all such medication will need to be discontinued for the course of the study. The withdrawal of medication will occur in accordance with previous trials of psilocybin for depression from our Centre (25, 27) and within the guidelines set out by the Royal College of Psychiatrists (90).

#### Screening and Consent

The screening procedure will involve both telephone/video-call and in-person screening. Potential participants will be provided with a summary information document followed by a full participant information document prior to providing consent. Initial consent to take part in the screening process will occur during the first screening call. Full study consent will be obtained at the in-person screening visit. Due to the centrality of AN in self-identity and personal values, capacity to provide informed consent may be compromised when making treatment decisions (91). In line with Park and colleague's Oxford Neuroethics Framework for DBS in AN (91), capacity to consent will be assessed at screening using the MacArthur Competence Assessment Tool for Clinical Research (MacCAT-CR) (92, 93).

Potential participants will also be asked to identify a support person such as a family member or close friend, and this person will be contacted by the study team. Consent will be obtained from the support person to (1) provide support during the study (required for the potential participant's participation), and (2) take part in a support-person sub-study (optional). See the section Support Person Involvement for further information.

#### Study Visits

**Figure 1:**
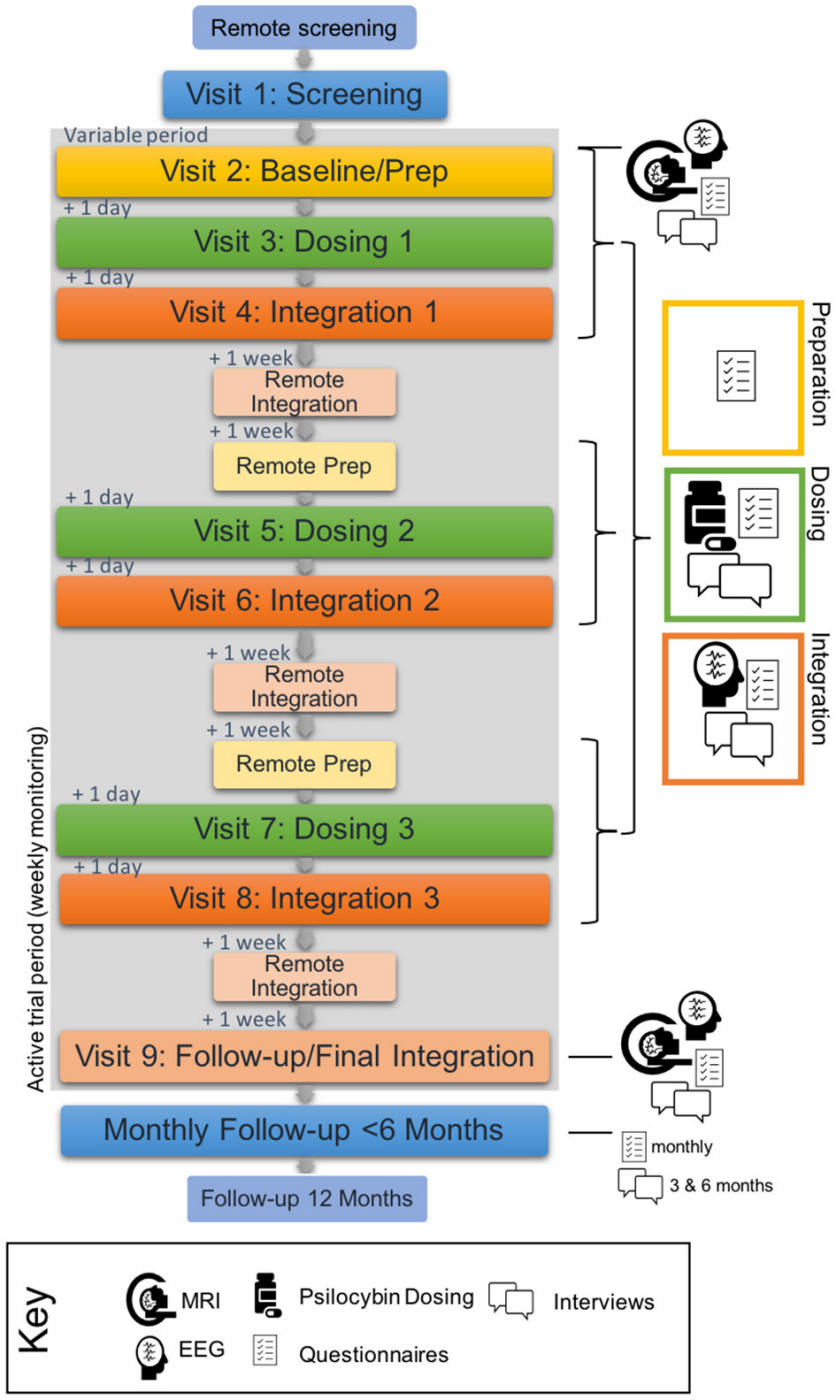
Study timeline. Grey background represents the active trial period. Blue represents pre- and post-trial periods. Yellow, green and orange represent preparation, dosing and integration respectively. Time intervals between each study day (remote or visit) for the active period are included on the left of the figure.

The timeline of the study visits can be found in Figure 1 and an overview of all study measures and activities can be found in Table 2. Over a period of 6 weeks, participants who are deemed eligible at screening will partake in eight study visits, including three psilocybin dosing sessions of up to 25 mg. Across these eight visits, there will also be two MRI scans, five EEG recordings and a range of psychological measures (questionnaires and interviews). There will be a follow-up period of 12 months following the final study visit, with monthly data collection points for the first 6 months.

**Table 2:** Schedule of study activities.

**Measures**	**Screening**	**6-Week study period**					**Monthly follow-up**
		**Baseline^#^**	Prep days^*^	**Dosing days**	**Integration days**	**6-Week endpoint^#^**	
**Enrolment**							
Informed consent	x						
MacCAT-CR	x						
Physical examination (incl. bloods and ECG)	x						
Psychiatric assessment	x						
MINI	x						
**Key study activities**							
Psilocybin dosing				x			
MRI		x				x	
EEG		x			x	x	
Support person contact	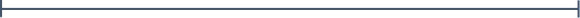						
GP and ED team contact	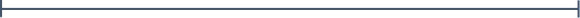						
Eating habits monitoring	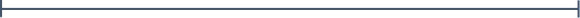						
Mood monitoring	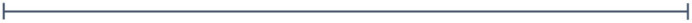						
Physiological measures	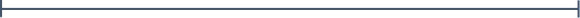						
Adverse events	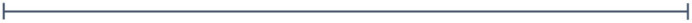						
**Primary & key secondary outcomes**							
RMQ	x	x	x			x	x
EDE (interview)		x				x	x^∧∧^
EDE-Q	x	x	x			x	x
**Psychological outcome measures**							
PHQ-9	x	x	x			x	x
BDI-II	x	x	x			x	x
WEMWBS		x	x			x	x
MPFI		x	x			x	x
b-EAQ		x			x	x	
Y-BCEDS		x				x	
CIA		x				x	x
RRS-ED		x				x	
STAI-T		x				x	
WCS		x	x			x	
SCS		x	x			x	x
EES		x	x			x	x
CFS		x	x			x	x
P-CAN		x	x			x	x
IDEA		x				x	x
MAIA		x				x	
Openness		x				x	
FSCS		x				x	
CEAS		x	x			x	
IUS		x				x	
Self-constructed items		x				x	
Support person questionnaire (self-constructed)		x				x	
Exploratory outcome measures		x	x			x	x
**Acute & integration measures**							
ASC				x			
CEQ				x			
MEQ				x			
EBI				x			
PMQ				x			
SM-B				x			
Self-constructed acute measures				x			
Self-constructed integration measures					x	x	x
COE						x	x
**Predictors**							
ACE		x					
Credibility/expectancy		x					
SSS		x					
MODTAS		x				x	
STAR			x				
STAI-S				x			
PPS				x			
**Self-constructed interviews**							
Recovery interview		x				x	
(R)EB-Q			x	x		x	
Support-Person interview						x	
Final interview							x^∧^
**Behavioural measures**							
TMT		x				x	x^∧^
WCST		x				x	x^∧^
LO-FPT		x	x			x	x^∧∧^
HRD		x				x	

# *remote measures will be collected within 1 week of study visit*.

* *Prep day measures before dosing day 1 are included in the baseline survey*.

*^∧^measures followed by ^∧∧^ are only included at 3 & 6 months. Measures followed by ^∧^ are included only at 6 months*.

*MacCAT-CR, MacArthur Competence Assessment Tool for Clinical Research (92); MINI, Mini international neuropsychiatric interview (94); RMQ, readiness and motivation questionnaire (95); EDE, eating disorder examination (96); EDE-Q, eating disorder examination questionnaire (96); PHQ-9, patient health questionnaire-9 [(97), p. 9]; BDI-II, Beck Depression Inventory II (98); WEMWBS, Warwick-Edinburgh Mental Wellbeing Scale (99); MPFI, Multidimensional Psychological Flexibility Inventory (100); b-EAQ, brief experiential avoidance questionnaire (101); YBC-EDS, Yale-Brown Cornell Eating Disorder Scale (102); CIA, Clinical impairment inventory (103); RRS-ED, Ruminative response scale for eating disorders (48); STAI-T, state trait anxiety inventory trait subscale (104); WCS, Watts connectedness scale (self-constructed); SCS, self-compassion scale (105); EES, experience of embodiment scale (106); Cognitive Flexibility Scale (CFS) (107); P-CAN, Pros and Cons of Anorexia Scale; IDEA, Identity in Eating disorders scale (78); MAIA, Multidimensional Assessment of Interoceptive Awareness (108); FSCS, Function of self-criticism/attacking scale (109); CEAS, Compassionate Engagement and Action Scale–self compassion and compassion for others subscale (110); IUS, Intolerance to Uncertainty Scale (111); ASC, altered states of consciousness questionnaire (112); CEQ, challenging experiences questionnaire (113); MEQ, mystical experiences questionnaire (113); EBI, emotional breakthrough inventory (41); PMQ, Psychedelic Music Questionnaire; SM-B, State of Mindfulness- body subscale (114); COE, Centraility of Events Scale (115) ACE, adverse childhood events (116); SSS, short suggestibility scale (117); MODTAS, Modified Tellegen Absorption Questionnaire (118); STAR, scale to assess the therapeutic relationship (119); STAI-S, state trait anxiety inventory state subscale (104); PPS, psychedelic predictor scale (self-constructed); (R)EB-Q, relaxed embodied beliefs questionnaire (self-constructed); TMT, trail making test (120); WCST, Wisconsin card sorting task (121); LO-FPQ, Leeds Oxford food preference questionnaire (122); HRD, heartrate discrimination tasks (123)*.

##### Baseline and 6-Week Follow-Up

Baseline and follow-up study measures will be collected both in person and remotely (via video-call and the online survey platform Alchemer). The baseline and 6-week follow-up visits (visit 2 and 9, respectively), will be run similarly and will include MRI and EEG. The MRI scanning procedure will take ~90 min, with the scan itself lasting 60 min. EEG recording will last ~40–60 min. The baseline day will also include a meeting with the guide team to prepare for the first dosing session the following day (more information below). The 6-week follow-up day will also include a final integration meeting with the guide team.

##### Psilocybin-Assisted Therapy

Each participant will be paired with two “guides” whom they will work with for the duration of the trial. In all cases, the guide pair will include a therapist and/or psychiatrist. Preparation will begin with two remote calls before an in-person session on the baseline day (visit 2). Preparation sessions for subsequent dosing days will primarily be conducted remotely, but can be conducted in-person if this is deemed appropriate and preferrable by the participant and their guides.

Dosing days (visits 3, 5, and 7) will typically last ~8 h, with the acute effects of the drug lasting 4–6 h. A medical professional will be on site for the duration of the dosing sessions and will approve the participant for discharge at the end of the day. Participants will be encouraged to utilise optional overnight accommodation the night prior to and following dosing. Those who live near the study centre may choose to go home; in which case, a taxi will be provided.

In-person integration visits will occur the day after dosing (visits 4, 6, and 8). The integration itself is anticipated to last 1–3 h, and will be followed by an EEG. Remote integration calls will take place 1 week after dosing.

Consistent with previous studies of psilocybin-assisted therapy, dosing will take place in a therapeutic environment. Participants will be encouraged to lie in a semi-reclined position and will be provided with an eye mask and headphones through which a specially designed music playlist is delivered.

The concomitant psycho-therapeutic models for psychedelic-assisted therapy have historically been informed by humanistic and psycho-analytic approaches, however, there has been growing interest in incorporating other evidence-based therapies into the psychedelic assisted model, such as Cognitive Behavioural Therapy [CBT (22, 124)] Acceptance and Commitment Therapy [ACT (125–127)], Motivational Interviewing (128), and Emotion-Focused Family Therapy [EFFT (129)]. There is also growing support for manualisation of adjunct therapeutic approaches (125, 126, 128). The manual for the current trial, which incorporates various approaches, will be made available upon completion. It is hoped that it will be useful in future trials and will continue to develop with the burgeoning field of psychedelic-assisted therapy.

#### Support Person Involvement

Given the complex and systemic nature of AN, it is highly recommended that the family and support networks be involved in the treatment process where appropriate (130). The involvement of key support people is also recognised to improve outcomes for all involved (131–134). Additionally, we recognise the important role that the support person will play in facilitating long-term change and the transition back into usual care once the 6-week trial is complete.

As part of the study, the participant's identified support-person will be actively involved in the study process. With prior consent from the support person (a necessary condition of participant's involvement in the trial), we will make contact with them prior to their respective participant starting the trial and 1 week after each dose to provide support, guidance and study-specific resources. Participants will be strongly encouraged to bring their support person with them to in-person visits to escort them between study activities. There will be scope for a participant's support person to enter the therapy room at the end of a session (preparation, dosing, integration), however they will not be present while the participant is still experiencing the acute drug effects, or at any point where this may impact the therapeutic intention of the trial, including study measures. Participants will always be consulted before inviting a support person to join a session.

We will also invite support people to take part in an optional Next-Of-Kin sub-study. This will consist of the completion of a questionnaire at baseline and at the 6-week endpoint, as well as a semi-structured interview at the end of the active 6-week trial period. Both the self-constructed questionnaire and interview have been designed to explore the support person's perspective on their loved one's eating disorder, their relationship with their loved one, and how this may have changed over the course of the trial.

#### General Analysis Strategy

##### Efficacy Outcomes

As there are no prior data on psychedelic-assisted therapy in AN and given that it is well-known that AN psychopathology can take time to shift, it seems important to focus on both short-term response to treatment (i.e., over the course of the 6-week trial), and long-term changes in psychopathology (i.e., over the follow-up period). It is not uncommon for pilot studies to define more than one primary outcome, especially when one such measure is clinical and the other is not, and the goal of the study is to assess response to treatment effects and variance (83, 84). There will be no correction for multiple comparisons as this is an exploratory study and no confirmatory claims will be made. We will report significance testing but note that effect sizes, confidence intervals and trends will be more meaningful overall in determining the trajectory of future studies.

For all psychological outcomes, treatment effects will be established by comparing baseline measures with the 6-week endpoint and/or monthly for a follow-up period of 6 months and at 12 months. Where relevant, further analyses will be conducted between dosing sessions. These analyses will largely consist of frequentist analyses such as analysis of covariance (ANCOVA) by timepoint controlling for baseline measures where relevant, or linear mixed models.

Predictors of treatment response and measures of the acute experience will be incorporated in mediation/moderation analyses (e.g., path analysis or structural equation modelling). Where parametric assumptions do not hold, non-parametric alternatives will be employed. Bayesian approaches will also be flexibly used to supplement frequentist hypothesis testing and to explore the relative evidence for a given effect.

##### Feasibility Outcomes

Consistent with our feasibility goals, we will also report recruitment and retention rate^5^. A consort diagram will be presented displaying participant retention at each phase of the recruitment, screening and data collection process. We will report the numbers of individuals provided with participant information sheets who subsequently decided not to participate in the trial, number that underwent telephone screening and number of drop-outs. For full transparency on inclusivity, information on the demographics and reason for exclusion will be reported from the screening dataset.

The level and pattern of the missing data in baseline variables and outcome measures will be reported. Where relevant, the potential causes for missing data will be investigated and reported.

Reported Adverse Events (AEs) will be recorded for the duration of the study. AEs will be classified by the clinical team based on severity (mild = no interference with daily activities; moderate = some interference with daily activities; severe = prevents daily activities), whether they are expected or unexpected, and causality (related, possibly related, or unrelated to the IMP). AE summary tables will display the total number of participants reporting an AE, the percentage of participants (%) with an AE, and the number of events (E) reported. Serious AEs (SAEs) and Serious Adverse Reactions (SARs) are defined as any untoward medical occurrence or effect that at any dose (i) results in death, (ii) is life-threatening, (iii) requires hospitalisation, or (iv) results in persistent or significant disability or incapacity. All SAEs and SARs will be reported to the PI, sponsors, and regulatory bodies within 24 h of the study team being made aware of the event.

#### Study Measures

##### Primary Psychological Outcome Measures

The two primary outcome measures are the Eating Disorder Examination (EDE) and the Readiness and Motivation Questionnaire (RMQ) (Table 3).

**Table 3:** Primary and key secondary outcomes.

**Measure**	**Timepoints for analysis**
**Primary outcome**	
Eating disorder examination (EDE) global (96)	Baseline−6-month follow-up Baseline−3-month follow-up Baseline−6-week follow-up
Readiness and motivation questionnaire (RMQ) pre-contemplation (95)	Baseline−6-week follow-up Comparison between dosing days
**Key secondary outcomes**	
Eating disorder examination questionnaire (EDE-Q) global (96)	Baseline−6-week follow-up Comparison between dosing days
RMQ pre-contemplation & EDE global	Change in RMQ from baseline−6 weeks as a predictor of change in EDE global across the 6-month follow-up

The investigator-led EDE and it's self-report counterpart, the Eating Disorder Examination Questionnaire (EDE-Q) are the most widely used indices of eating disorder psychopathology in research settings (96). Both measures assess the frequency and severity of eating disorder cognitions and behaviours over the past 28 days. The EDE interview, which is administered by a trained researcher, gives a detailed, objective assessment of psychopathology and is widely viewed as the “gold standard” measure. Because it provides a detailed measure of the range and severity of eating disorder features it is used in most treatment studies and in many other in-depth investigations of eating disorder psychopathology. The EDE will be administered four times throughout the study: baseline, 6-week endpoint, 3-month and 6-month follow-up. Change in EDE (global score) will act as primary/key secondary outcome for this study, which will be analysed (1) from baseline to 6-month follow-up, (2) from baseline to 3-month follow-up, and (3) from baseline to 6-week follow-up. Secondary analysis will be performed on the four subscales (restraint, eating concerns, shape concerns, weight concerns).

The EDE-Q is a self-report questionnaire based upon the EDE interview and may be used when it is impracticable or undesirable to employ the interview. Here, we use the EDE-Q as supplementary to the EDE as it is more practical for more regular and remote administration. The EDE-Q will be measured bi-weekly for the duration of the trial (with adjustments made to reference the relevant time-period), monthly for the first 6 months of the follow-up period, and once more at 12-months. A key secondary analysis will be performed on EDE-Q global scores between the dosing sessions. Secondary analysis will be performed on the four subscales (restraint, eating concerns, shape concerns, weight concerns).

Motivation for recovery is a widely acknowledged barrier to treatment for AN and an essential first step of any effective treatment (64, 70, 79, 81). The Readiness and Motivation Questionnaire (RMQ) provides total readiness scores (for pre-contemplation, action, internality, and confidence), as well as readiness scores for four domains of eating disorder psychopathology (cognition, restriction, bingeing, compensatory strategies) in reference to the past 2 weeks. Both the RMQ and the interview upon which it is based (the Readiness and Motivation Interview) have been demonstrated as reliable predictors of behaviour change (95, 135–138), with a decrease in pre-contemplation score most sensitive to change in AN (95, 138–140). The RMQ will be measured bi-weekly for the duration of the trial, monthly for the first 6 months of the follow-up period, and once more at 12-months. The primary comparison will be pre-contemplation scores between baseline and the end of the active trial period (6-week follow-up), and between dosing days. Secondary analysis will also explore changes in other RMQ subscales, and additional time points.

Given the interplay between readiness for recovery and changes in eating disorder psychopathology, a final key secondary analysis will assess whether changes in RMQ during the trial period predict change in EDE/EDE-Q over the 6-month follow-up period.

##### Secondary Psychological Outcome Measures

Secondary psychological measures will explore other areas of psychopathology and impairment related to AN, including day-to-day impairment [Clinical Impairment Assessment (CIA) (103)], rumination [Ruminative-Response Scale for Eating Disorders (RRS-ED) (48)], and preoccupation and rituals [Yale-Brown Cornell Eating Disorder Scale (YBC-EDS) (102)]. Given evidence for the possible transdiagnostic efficacy of psilocybin (20), and high rates of comorbidity in AN (141), we will also measure changes in depression symptomology [Beck Depression Inventory 2 (BDI-II) (98), Patient Health Questionnaire (PHQ-9) (97)], and anxiety [State-Trait Anxiety Inventory- Trait (STAI-T) (104)].

As discussed in the PPI focus groups, recovery from AN does not only pertain to changes in clinical symptomology, but involves *rediscovery* and *reconnection* with one's self, and one's values. To measure changes in the broader impact of AN on participants' lives we have included measures of AN beliefs and values [Pros and Cons of Anorexia Scale (P-CAN) (75)], AN identity [Identity in Eating disorders scale (IDEA) (78)], embodiment [Experience of Embodiment Scale (EES) (106), Multidimensional Assessment of Interoceptive Awareness (MAIA) (108)], self-compassion [the Self-Compassion Scale (SCS) (105)], intolerance to uncertainly [Intolerance to Uncertainty Scale (IUS) (111)] and cognitive flexibility [Cognitive Flexibility Scale (CFS) (107)]. Based on previous psychedelic trials, we will also measure changes in more generalised constructs that have been shown to shift following psychedelic-assisted therapy and that have an important role in recovery from AN. This includes psychological well-being [Warwick-Edinburgh Mental Well-being Scale (WEMWBS) (99)], psychological flexibility (Multidimensional Psychological Flexibility Inventory (MPFI) (142), experiential avoidance [brief experiential avoidance questionnaire (BEAQ) (101)], and connectedness (Watt's Connectedness Scale).

Acknowledging that recovery from AN is a unique and personalised journey, and the exploratory nature of the trial, we have also included a variety of self-constructed qualitative/quantitative interviews. These measures explore topics such as individual definitions of recovery (the Recovery Interview), changes in embodied core beliefs [the (Relaxed) Embodied Beliefs Questionnaire ((R)EB-Q)], and experience of psilocybin therapy (6-month interview).

Finally, given the role of the acute experience in predicting psychological outcome following a psychedelic experience (40, 143), various measures of the acute experience have been included (Table 2). While there is a current lack of validated measures of integration, a mixture of validated and self-constructed measures of integration have also been included.

##### Neuroimaging

We will perform brain imaging (MRI) at baseline (visit 2), and 6-week follow-up (visit 9). Our primary imaging outcome measures will reflect functional (fMRI) brain changes and will include a change in task-based BOLD response and a change in resting-state activity and connectivity. As secondary measures, we will also assess structural changes (morphometry, cortical thickness and diffusion imaging).

Electroencephalography (EEG) will be collected at baseline (visit 2), integration 1 (visit 4), integration 2 (visit 6), integration 3 (visit 8), and 6-week follow-up (visit 9). Resting-state data will be collected at all time points. As a key index of induced changes in neuroplasticity, we will include the visual Long-Term Potentiation (LTP) paradigm (144) on integration days. The roving Mismatch Negativity (MMN) paradigm will also act to supplement neuroplasticity findings (145, 146).

##### Behavioural and Other Additional Measures

The Wisconsin Card Sorting Task [WCST, (121)] and Trail Making Test [TMT, (120)] will be conducted as measures of cognitive flexibility at baseline (visit 2), 6-week follow-up (visit 9) and during the 6-month follow-up. Interoceptive accuracy and precision will be assessed using the Heartrate Discrimination Task [HRD, (123)] at baseline (visit 2) and follow-up (visit 9). The Leeds-Oxford Food Preference Task [LO-FPT, (122)] will be employed to detect changes in preference for low-fat, high-fat, low-energy, and high-energy foods at baseline (visit 2), preparation days 2 and 3 (visits 4 and 6), follow-up (visit 9), and at the 3- and 6-month remote follow-ups. Physiological measurements (e.g., respiratory rate, heartrate variability, and accelerometery) may be collected from consenting participants throughout the study.

#### Data Management

Data will be managed as per the Imperial College Data Management Standard Operating Procedures and a study-specific data management plan (authored by MS). Data management will be overseen by the trial co-ordinator (MS).

The GDPR-compliant Psychedelic Survey platform (psychedelicsurvey.com) is an external software that was created for our centre and will be used to deliver remote survey measures. Participants will receive links to the surveys via email, to be completed on the separate survey hosting platform Alchemer, where we will store our data (to GDPR standards). In previous studies, this was found to be an efficient way to optimise collection and safe storage of questionnaire data, while also reducing human error, and so this procedure will be repeated for this trial. The WCST, TMT and LO-FPT will be hosted on the online behavioural task platform Cognitron, developed by the Imperial Computational, Cognitive, and Clinical Neuroimaging Laboratory. Participants will be delivered remote links to these tasks via the same Psychedelic Survey emails.

The study will be monitored internally by the Centre for Psychedelic Research, by Imperial College Healthcare NHS Trust, by Imperial College RGIT (sponsors) and it may also be audited by the MHRA. Any trial-related regulatory inspections will be permitted and direct access to source data and documents will be provided.

#### Participant Safety and Monitoring

Participants' mood and eating habits will be monitored weekly from enrolment until the end of the 6-week active study phase using self-report measures administered via the remote survey platform. On weeks where no study visits are scheduled during the active study period, phone or video calls will allow the study team to check-in with the participant. Halfway through the trial, we will report back to the care team on any changes in the participants well-being or changes that are deemed clinically significant. Additionally, we will contact the participant's identified support person at set timepoints throughout the study. The study team will be contactable by the participant's care team and support person throughout the active study phase.

We will record participant's weight on five occasions throughout the study: screening (visit 1), baseline (visit 2), dosing day 2 (visit 5), dosing day 3 (visit 7), and 6-week follow-up (visit 9). This will be discussed with participants during the screening and preparation process to best manage any anxiety with weight checks. If rapid weight loss occurs (>2 kg in a month) participants will be withdrawn due to medical instability, and this will be reported to the relevant healthcare professional(s). Specific consent is obtained for weight information to be shared between the study team and the participants eating disorder team should this be required. Should there be any other significant deterioration in the participant's physical or mental health we will liaise with the relevant health professionals to determine whether is it safe, and in the participants best interests, to continue in the study. This will occur in discussion with the participant and the extended research team, if relevant.

#### IMP Management

A Schedule 1 licence has been obtained from the UK Home Office. Psilocybin has been supplied by Compass Pathways. Manufacture was performed by Onyx and encapsulation was performed by Catalent (formerly Juniper) pharmaceuticals. Bottling and labelling were performed by Fisher pharmaceuticals. Good Manufacturing Practise (GMP) has been maintained at all stages of manufacture. The IMP will be stored and dispended by Invicro LLC.

#### Dissemination

The results of this study will be published in academic journals and presented in both the academic and public domain, including at scientific conferences and in the media in public engagement forums. Patient confidentiality will be maintained in all of the above.

#### Ethics Statement and Trial Registration

This is an investigator-initiated, university-sponsored trial. This trial has received a favourable opinion from Brent National Research Ethics Service (NRES) and is sponsored by Imperial College London's Research Governance and Integrity Team (RGIT, formerly the Joint Research Compliance Office; JRCO). It has been reviewed and approved by the Health Research Authority (HRA) and Medicines and Healthcare products Regulatory Agency (MHRA). All staff have undergone Good Clinical Practise (GCP) training. The study has been adopted by the National Institute of Health Research (NIHR) Clinical Research Network (CRN) and has been registered on clinicaltrials.gov (NCT04505189). All study sessions will take place at the NIHR-funded Imperial College Research Facility (ICRF).

## Conclusions

Here we have presented a trial protocol for an innovative pilot study assessing psilocybin-assisted therapy as a treatment component for AN. While this trial is one of many exploring psychedelics in mental health treatment, we wish to emphasise how incorporating the voices of those with lived experience has not been beneficial for adapting the psychedelic-therapy approach to this population, but has also been a rewarding experience for researchers and focus group attendees alike. We encourage the integration of PPI initiatives into future psychedelic research, and guidance for doing so has been presented by Close et al. (59).

It is hoped that by presenting our protocol in this format, we will improve the transparency and methodological rigour of the trial which, in turn, will enhance its impact. The insight gained from this pilot study will not only shed light on the potential of psilocybin-assisted therapy in the treatment of AN but will also provide a framework for a more efficient, ethical, and efficacious RCT in the future.

## Ethics Statement

The studies involving human participants were reviewed and approved by Brent National Research Ethics Service. The patients/participants will provide their written informed consent to participate in this study.

## Author Contributions

Study conceived by MS, RC-H, and DJN. Protocol written by MS, RC-H, DE, and DJN. Study documentation written by MS. Regulatory approvals obtained by MS, HD, DE, DJN, and RC-H. Supervision and guidance provided by RP, DEN, AL, TW, and TR. Statistical analysis plan by MS and AB. PPI facilitated by MS, HD, KA, and FM. Trial coordination by MS and HD. Data collection and analysis by MS, HD, RP, FM, KA, and JD. Trial clinical team FM, KA, JD, RP, and TR. All authors contributed to the article and approved the submitted version.

## Funding

This study is funded by the funders of Imperial College London's Centre for Psychedelic Research (https://www.imperial.ac.uk/psychedelic-research-centre/funding-partners/), with specific support from The Nikean Foundation. Psilocybin has been supplied by COMPASS Pathways. HD was funded by the President's Ph.D. Scholarships.

## Author Disclaimer

The views expressed are those of the author(s) and not necessarily those of the NHS, the NIHR or the Department of Health and Social Care.

## Conflict of Interest

RC-H reports receiving consulting fees from COMPASS Pathways, Entheon Biomedical, Mydecine, Synthesis Institute, Tryp Therapeutics, and Usona Institute; DE receiving consulting fees from Field Trip and Mydecine and Entheon Biomedical; and DJN receiving consulting fees from Awakn, H. Lundbeck, and Psyched Wellness, advisory board fees from COMPASS Pathways, and lecture fees from Takeda Medical Research Foundation and owning stock in Alcarelle. The remaining authors declare that the research was conducted in the absence of any commercial or financial relationships that could be construed as a potential conflict of interest.

## Publisher's Note

All claims expressed in this article are solely those of the authors and do not necessarily represent those of their affiliated organizations, or those of the publisher, the editors and the reviewers. Any product that may be evaluated in this article, or claim that may be made by its manufacturer, is not guaranteed or endorsed by the publisher.

## Footnotes

^1^There is debate over the most appropriate treatment target and avenue for Severe Enduring Anorexia Nervosa (SEAN); for example, whether quality of life is a more appropriate target than weight restoration, whether compulsory treatment should be repeatedly enforced, or whether, in some cases, palliative care is instead justified (16, 17).

^2^Bornemann J, Close JB, Spriggs MJ, Carhart-Harris RL, Roseman L. *Psychedelic Self-Medication for Chronic Pain: A Qualitative Investigation* (under review).

^3^While some have argued that AN is characterised by a lack of insight into the seriousness of the condition (69, 79, 80), it may be that, at least in some cases, this is rather a projection of the internal contradiction between simultaneously held beliefs (i.e., a projection of ambivalence) than a lack of insight *per se*.

^4^According to Thabane et al. (84), this is perhaps a more important consideration than sample size to ensure that outcomes are transferrable.

^5^It has been suggested that the large number of measures in our study may lead to increases in drop-out rates. However, across previous trials of similar design in participants with depression and healthy volunteers, we did not have any drop-out for this reason. Drop-out rates will nevertheless be an important feasibility measure prior to a large-scale RCT.
